# Synchronously Tailoring Strain Sensitivity and Electrical Stability of Silicone Elastomer Composites by the Synergistic Effect of a Dual Conductive Network

**DOI:** 10.3390/polym8040100

**Published:** 2016-03-31

**Authors:** Nanying Ning, Sishu Wang, Liqun Zhang, Yonglai Lu, Ming Tian, Tung W. Chan

**Affiliations:** 1 State Key Laboratory for Organic-Inorganic Composites, Beijing University of Chemical Technology, Beijing 100029, China; ningny@mail.buct.edu.cn (N.N.); luyonglai@mail.buct.edu.cn (Y.L.); 2 Key Laboratory of Carbon Fiber and Functional Polymers, Ministry of Education, Beijing University of Chemical Technology, Beijing 100029, China; wangsishu@139.com; 3 Department of Materials Science and Engineering, Virginia Polytechnic Institute and State University, Blacksburg, VA 24061, USA; tungwchan@163.com

**Keywords:** conductive polymer composites, tensile strain sensing, sensitivity, conductive filler network

## Abstract

The use of conductive polymer composites (CPCs) as strain sensors has been widely investigated. A wide range of strain sensitivities and high repeatability are vital for different applications of CPCs. In this study, the relations of the conductive filler network and the strain-sensing behavior and electrical stability under fatigue cycles were studied systematically for the first time based on the conductive polymethylvinylsiloxane (PMVS) composites filled with both carbon nanotubes arrays (CNTAs) and carbon black (CB). It was proved that the composites could be fabricated with large strain-sensing capability and a wide range of strain sensitivities by controlling the volume ratio of CNTA/CB and their amounts. Additionally, the CNTA/CB/PMVS composite with 3 vol % content of fillers showed high sensitivity (GF is 10 at 60% strain), high repeatability (the relative standard deviation (RSD) of the max *R*/*R*_0_ value is 3.58%), and electrical stability under fatigue cycles (value range of *R*/*R*_0_ is 1.62 to 1.82) at the same time due to the synergistic effects of the dual conductive network of CNTAs and CB. This could not be achieved by relying on a single CNTA or CB conductive network. This study may provide guidance for the preparation of high performance CPCs for applications in strain sensors.

## 1. Introduction

Conductive polymer composites (CPCs) have attracted a large amount of attention and been widely used in industry for applications such as electromagnetic interference (EMI) shielding, touch control switches and sensors [1,2,3] by virtue of easy fabrication methods, and the good electrical conductivity of conductive fillers. Recently, CPCs acting as sensors for strain [4], stress [5,6], temperature [7], and liquid [8] have been investigated. CPCs have a wide range of applications in strain sensing, including smart textile [9], health monitoring [10], and movement sensors [11]. Various and suitable resistance-strain sensitivities are desirable for different applications as required. In addition, the repeatability of the strain sensor is one index of the sensor. Excellent repeatability of the strain sensor contributes to reducing the measurement error.

The resistance-strain sensitivity is closely related to the formation of conductive network. The conductive network is mainly affected by the content and length to diameter ratio of conductive fillers, the dispersion of the fillers, and the filler-matrix interface. Therefore, the kind and content of fillers as well as the choice of matrix affect the strain-sensing behavior of composites greatly. Zhao *et al.* [12] studied the tensile strain-sensing behaviors of carbon black (CB)/polypropylene (PP) and carbon nanotubes (CNTs)/PP composites and found the responsivity increased in an exponential and linear fashion for CB/PP and CNTs/PP with the filler concentration just beyond the percolation region, respectively, and they explained the phenomenon by the tunneling theory. Dang *et al.* [13] observed that CNTs with a higher aspect ratio were preferred for a higher sensitivity in CNT/silicone rubber nanocomposites. Costa *et al.* [14,15] studied the effects of different filler contents and filler functionalizations of CNT/styrene–butadiene–styrene (SBS) composites on the electro-mechanical properties. Fathi *et al.* [16] found that the conductivities of the CB/low density polyethylene (LDPE) and CB/PP CPCs at CB concentrations near the high end of the percolation region were sensitive to the applied strain. Knite *et al.* [17] observed that, for CB/polyisopree CPC samples near the percolation transition region, a strong responsivity of electrical resistance change is obtained.

On the other hand, different strain sensitivities of composites are often required for different applications [18]. The control of sensitivity for strain sensors is a vital issue, and some methods such as the addition of secondary fillers [19], the selective localization of filler in the matrix [20], and the use of mixed and functionalized fillers [21] have been demonstrated to modify the strain sensitivity of CPCs. Witt *et al.* [22] prepared a conductive silicone rubber (SR) composite filled with both CNTs and CB, and the SR composite showed improvement in mechanical properties, high conductivity at a comparatively low concentration, and high sensitivity for tensile and compressive stress. Nevertheless, little attention has been paid to the repeatability of the conductive composites for strain sensors [12,23,24,25]; it is still a big challenge to prepare materials with high strain sensitivity and high repeatability at large strain. Additionally, the relations of the conductive filler network and the strain-sensing behavior and electrical stability have yet not been elucidated.

Owing to the advantages of flexibility and softness similar to those of the human skin and muscle, excellent biocompatibility and chemical stability, and no toxicity, SR is one of the candidates applicable to the production of human wearable sensors. In our previous study [26], we prepared elastomer composites with excellent elasticity and conductivity, and good mechanical properties, for the first time by using carbon nanotube arrays (CNTAs) as nanosprings. The special CNTAs were well dissociated into many bendable single CNTs with almost no change in length in polymethylvinylsiloxane (PMVS) with a very low viscosity and exhibited a nanospring effect. In this study, we used the same CNTA/CB/PMVS system. Three volume fractions of nanofillers, which represent the volume fractions corresponding to the start of the percolation threshold, the end of the percolation threshold, and that far exceeding the percolation threshold, were used for comparison to reveal the relationship of the formation of the conductive filler network and the strain-sensing behavior and electrical stability. We controlled the conductive filler network by choosing different filler contents and the volume ratio of CNTA/CB to try to achieve tunable sensitivity of composites, and eventually acquire composites with high sensitivity, high repeatability, and electrical stability under fatigue cycles.

## 2. Experimental Section

### 2.1. Materials

Polymethylvinylsiloxane (PMVS, *M*_n_ = 570 K) consisting of 0.12–0.2 wt % vinyl was purchased from China National Blue Star (Group) Co., Ltd., Beijing, China. Carbon nanotube arrays (CNTAs) were supplied by CNano Technology Co., Ltd., Beijing, China. Carbon black (CB, EC600-JD) was purchased from Akzo Nobel Corp (Tokyo, Japan). γ-(methacryloxy) proxyltrimethoxysilane (KH570, CH_2_=C(CH_3_)COOCH_2_CH_2_CH_2_Si(OCH_3_)_3_) was provided by Nanjing Crompton Shuguang Organosilicon Specialties Co., Ltd., Nanjing, China. The vulcanizing agent (2,5-dimethyl-2,5-bis(tert-butyl peroxy) hexane, DBPMH) was purchased from Akzo Nobel Corp, Changshu, China. The co-curing agent triallylisocyanurate (TAIC) was supplied by Hunan Liuyang Chemical Co., Ltd., Liuyang, China.

### 2.2. Preparation of Composites

CNTAs were first modified by KH570 (the loading level was 5 wt % of a CNTA) according to the method presented in reference [27]. For the CNTA/PMVS composites, PMVS and the modified CNTAs were first mixed by using a two-roll mill (XK-160, Zhanjiang Machinery Factory, Zhanjiang, China). The gap between the two rolls was adjusted to ~0.5 mm for high-shear blending, which was followed by the addition of the vulcanizer (weight ratio of 20:1). The optimum curing time T_90_+3 min was determined with an oscillating disc curemeter (MR-C3, Beijing Huanfeng Instrument Co., Ltd., Beijing, China), and the composite was vulcanized on a lab platen press (25t, Shanghai Rubber Machinery Factory, Shanghai, China) under 25-ton pressure. The vulcanization temperature was 170 °C. Then, the composite was vulcanized in a draught drying cabinet (DHG-9246A, Shanghai Jinghong Experimental Equipment Co., Ltd., Shanghai, China) under air atmosphere at 200 °C for 2 h. The same procedure was used to prepare CB/PMVS composites and CNTA/CB/PMVS composites. For the CNTA/CB/PMVS composites, CB was first mixed with PMVS before the above procedure. The composites were kept for 24 h under standard experimental conditions (23 ± 2 °C, relative humidity 50% ± 10%) before testing.

### 2.3. Characterizations

The volume resistivity of the composites with a high resistivity (≥10^4^ Ω) was measured by using a high resistance meter (PC68, Shanghai Precision & Scientific Instrument Co., Ltd., Shanghai, China). A DC Bridge (QJ84, Shanghai Zhengyang Instrument Factory, Shanghai, China) was used to measure the volume resistivity of composites with low resistivity according to Chinese standard GB/T 2439-2001. The size of the test samples was 10 mm × 100 mm × 2 mm. The current electrodes were connected to the entire width of the sample, and the voltage electrodes were placed on the sample to measure. The volume resistivity (*ρ)* was calculated by

*ρ = R × S/L* (1)

where *R* is the electrical resistance of the sample, *S* is the cross-sectional area, and *L* is the length of the sample. Five samples were tested for each composite and the average value was reported.

Mechanical property was measured by using a universal material testing machine (CMT4104, Shenzhen SANS Testing Machine, Shenzhen, China) in accordance with GB/T 528-1998. The dumbbell samples (thickness, 2.0 mm ± 0.2 mm) were tested at a crosshead speed of 500 mm/min. The same samples were used for the tensile strain-sensing tests. Composite films were clamped between a pair of steel electrodes, creating a gauge length of 20 mm. The resistance was measured with a Keithley 2450 Source Meter (Keithley Instruments, Inc., Cleveland, OH, USA). The samples were stretched in a SANS CMT4104 universal testing machine at a crosshead speed of 50 mm/min. The resistance measurement setup and tensile test machine were both interfaced with a computer to record the *R*/*R*_0_ (*R* is the resistance of the sample, and *R*_0_ is the initial resistance of the sample without load)-stress (σ)-strain (ε) relationship. At least five samples were tested for each experiment and representative results were presented. In order to study the strain-sensing repeatability of composites, 10 extension–retraction cycles were conducted. The program of cyclic deformation included stretching to 60% strain at 50 mm/min, and withdrawing at 50 mm/min to the initial length of 20 mm.

Fatigue tests were carried out on a fatigue-testing machine for rubber (MZ-4003, Jiangdu Mingzhu Testing Machine Factory, Yangzhou, China) under a fixed tensile strain of 30%. The size of samples was 10 mm × 100 mm × 2 mm, and the actual length of the measurement area was 50 mm. The numbers of cycles were 10^2^, 10^3^, and 10^4^. The volume resistance was measured after the sample was unloaded and placed under the standard experimental conditions for 30 min. For each experiment in this study, at least three specimens were tested for statistical analysis.

The network structure of the conductive fillers in the composites was characterized by using a Rubber Process Analyzer (RPA-2000, Alpha Technologies Co., Ltd., Akron, OH, USA). Samples were vulcanized at 170 °C and tested at 60 °C under a frequency of 1 Hz with the strain amplitude ranging from 0.28% to 400%. The dispersion of CB and that of CNTAs were observed by using an atomic force microscope (Multimode8, Bruker Daltonics Inc., Karlsruhe, Germany) and a transmission electron microscope (JEM-2100, JEOL, Tokyo, Japan), respectively. The working mode of the atomic force microscope (AFM) was Peakforce QNM. Morphological studies using a transmission electron microscope (TEM) were carried out under an accelerating voltage of 200 KV, and the samples were prepared from ultrathin sections.

## 3. Results

### 3.1. Electrical and Mechanical Properties

**Figure 1:**
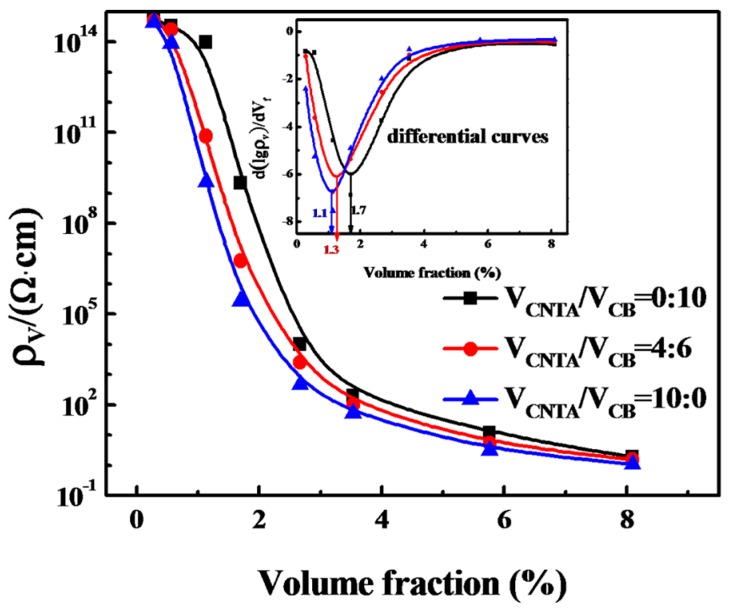
Volume resistivity against the filler volume fraction of CNTA/PMVS, CNTA/CB/PMVS, and CB/PMVS composites.

Many different contents ranging from 0.28 to 5.7 vol % of CB and CNTAs in PMVS were used to achieve the percolation threshold, at which the volume resistivity of the conductive composites decreases sharply owing to the fact that conductive fillers come to contact each other and lead to the formation of a conductive network in the matrix. From Figure 1, we can observe that the percolation threshold (*f*_p_) of CNTA composites is 1.1 vol %, whereas those of CNTA/CB composites and CB composites are 1.3 and 1.7 vol %, respectively. Therefore, three volume fractions of nanofillers, which represent the volume fractions corresponding to the start of the percolation threshold (1.5 vol %), the end of the percolation threshold (3 vol %), and that far exceeding the percolation threshold (6 vol %), are used for comparison. The results of the electrical and mechanical properties are shown in Figure 1 and Table 1.

Table 1 shows that, with the increase of the filler volume fraction and the volume ratio of CNTA/CB, the volume resistivity of the composites decreases from 1.2 × 10^11^ to 3.3 Ω·cm. At the filler content of 1.5 vol %, the conductivities of the three composites are very different. The CB/PMVS composite is still insulating, and the conductivity of the CNTA/PMVS composite is several orders of magnitude higher than those of the CNTA/CB/PMVS composite and the CB/PMVS composite. For composites with 3 vol % of fillers, the conductivity improve at least four orders of magnitude, and the volume resistivities are lower than 10^4^ Ω·cm. As the filler content comes to 6 vol %, a more complete network structure is formed for all the composites, and the composites are highly conductive.

With the increase of the filler volume fraction and the volume ratio of CNTA/CB, the tensile strength increases from 0.4 to 5.4 MPa. At the same filler volume fraction, as the volume ratio of CNTA/CB increases, the strain to failure decreases. It was noted that the strain to failure of all composites was able to reach above 78% strain, illustrating that the composites can bear large strain when used for strain sensing.

### 3.2. Strain-Sensing Behavior of Composites

#### 3.2.1. Resistance-Strain Sensitivity

**Figure 2:**
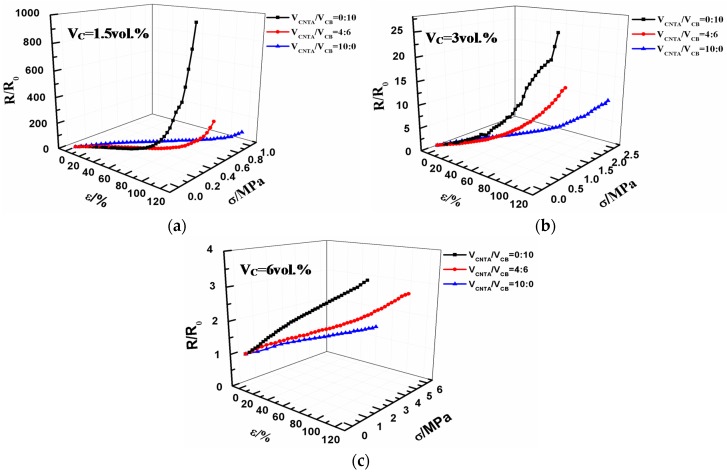
Strain-sensing behavior measurement for CB/PMVS, CNTA/CB/PMVS, CNTA/PMVS composites at different volume fractions of fillers: (**a**) 1.5 vol %, (**b**) 3 vol %, and (**c**) 6 vol %.

Tensile sensitivity is defined as the dependence of the resistance ratio *R*/*R*_0_ on the applied tensile strain ε. *R* and *R*_0_ refer to the resistance and initial resistance, respectively. The *R*/*R*_0_-stress (σ)-strain (ε) relationship is given in Figure 2.

Figure 2 reveals the correspondence among tensile strain, stress, and the change of resistance. Different composite materials filled with CB or CNTAs were investigated to review tensile sensitivity of our composites under uniaxial strain. The resistance of all the composites increases gradually with increasing strain, in agreement with previous reports [22]. It was noted that all composites samples were able to be elongated to more than 60% strain before rupture, indicating that the composites could be fabricated with large strain-sensing capability. Tensile sensitivity of composites largely depends on the filler volume fraction. Lower filler volume fraction contributes to higher sensitivity. When the filler volume fraction is 1.5 and 3 vol %, *R*/*R*_0_ increases in an exponential fashion. The filler volume fraction comes to 6 vol %, *R*/*R*_0_ increases almost linearly with the strain. As indicated by the slope coefficient of *R*/*R*_0_-ε curve, lower volume ratio of CNTA/CB in carbon fillers under the same filler volume fraction contributes to higher sensitivity.

As is well known, gauge factor (GF) can be introduced to quantify sensitivity for strain sensors. GF is the instant ratio of relative change in electrical resistance to the mechanical strain.

GF *=* d(*R/R_0_*)/dε (2)

*R* and *R_0_* refer to the resistance and initial resistance, whereas ε represents strain. To achieve high sensitivity, a higher value of GF is desirable.

**Figure 3:**
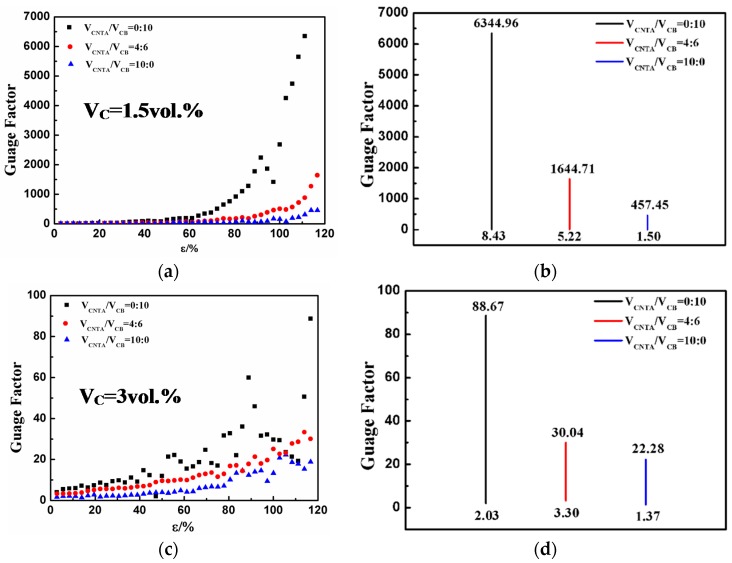

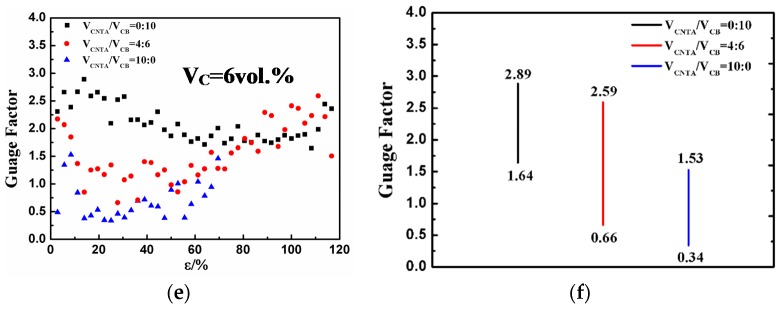
Gauge factor as a function of strain: (**a**) 1.5 vol %, (**c**) 3 vol % and (**e**) 6 vol %; range of sensitivity: (**b**) 1.5 vol %, (**d**) 3 vol % and (**f**) 6 vol %.

The GF of the composites in the current study can be calculated using the data shown in Figure 2. In Figure 3, it can be seen that the CB/PMVS composite with 1.5 vol % of fillers is obviously the most sensitive, with gauge factors of around 6345 at 110% strain and 192 at 60% strain. Higher volume ratio of CNTA/CB and the filler volume fraction contribute to lower GF. For the CNTA/CB/PMVS and CNTA/PMVS composites with 1.5 vol % of fillers, gauge factors correspond to 74 and 35 at 60% strain, respectively. At 60% strain, gauge factors of CB/PMVS, CNTA/CB/PMVS, and CNTA/PMVS composites with 3 vol % of fillers correspond to 19, 10 and 5. For composites with 6 vol % of fillers, gauge factors are all below 3. For composites with 1.5 and 3 vol % of fillers, GF has an upward trend with increasing strain. On the contrary, as the filler volume fraction comes to 6 vol %, GF changes slightly in a very small range. As shown in Figure 3, a wide range of tunable GF of 1.5 to 6345 (at 110% strain) and GF of 1 to 192 (at 60% strain) are obtained by using different filler contents and volume ratios of CNTA/CB in the current study. With the filler volume fraction of 1.5 and 3 vol %, the composites show high sensitivity for tensile strain.

#### 3.2.2. Repeatability of Dynamic Strain-Sensing Behavior

**Figure 4:**
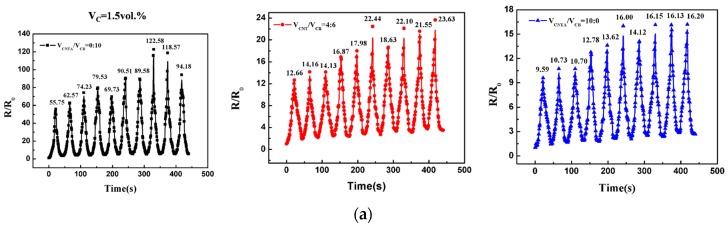

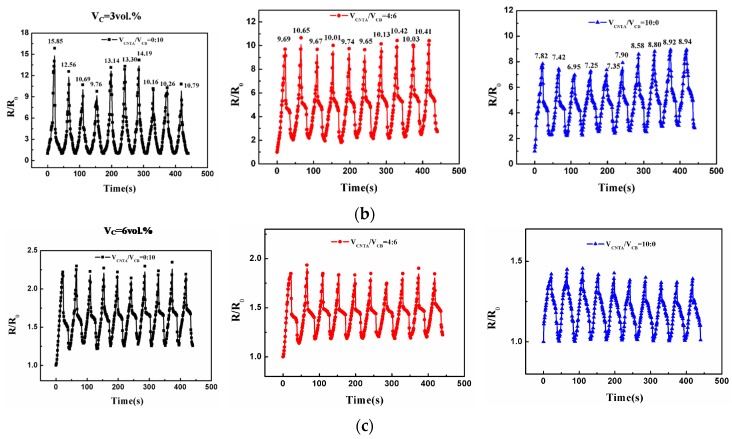
Relative R-time relationship for composites of 1.5 vol % (**a**), 3 vol % (**b**), and 6 vol % (**c**) fillers.

As the application of strain sensing often requires reversible strain loadings, composites were subjected to 10 cycles of extension–retraction strain. The maximum strain is 60%, and the relative resistance (*R*/*R*_0_) is plotted from zero-strain against time. Dynamic strain-sensing behavior is shown in Figure 4. It was noted that the R/R_0_ generally increases with increasing strain and decreases with decreasing strain. This can be defined as positive strain effect.

As shown in Figure 4a, when the filler volume fraction is 1.5 vol %, a fluctuation in *R*/*R*_0_ peak is observed in the dynamic cycles. For the CB/PMVS composite with 1.5 vol % of fillers, the value of the max *R*/*R*_0_ increases to about 2.2 times after 8 extension-retraction cycles, and then decreases to 1.7 times of the initial value of the max *R*/*R*_0_, and the relative standard deviation (RSD) of the max *R*/*R*_0_ value is 25.82%. For the CNTA/CB/PMVS and CNTA/PMVS composites, the value of the max *R*/*R*_0_ increases to about 1.9 and 1.7 times of the initial value of the max *R*/*R*_0_, and the RSDs of the max R/R_0_ value are 21.34% and 18.84%, respectively. As shown in Figure 4b, for the CB/PMVS and CNTA/PMVS composites with 3 vol % of fillers, a fluctuation in *R*/*R*_0_ peak is observed first, and then the strain-sensing behavior remains repeatable after 7 extension-retraction cycles, and the RSDs of the max *R*/*R*_0_ value are 16.91% and 9.49%, respectively. For the CNTA/CB/PMVS composite with 3 vol % of fillers, the RSD of the max *R*/*R*_0_ value is 3.58%, illustrating the dynamic strain-sensing behavior of the composite is repeatable. As shown in Figure 4c, when the filler volume fraction comes to 6 vol %, the *R*/*R*_0_ becomes recoverable, and the strain-sensing behavior remains repeatable, as the RSDs of the max *R*/*R*_0_ value of all the composites are less than 2.7%.

### 3.3. Electrical Stability under Fatigue Cycles

**Figure 5:**
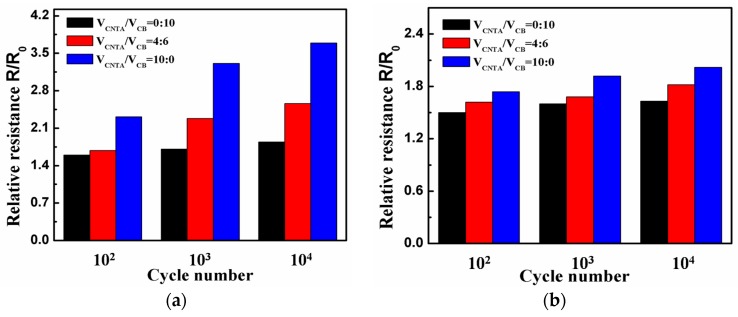

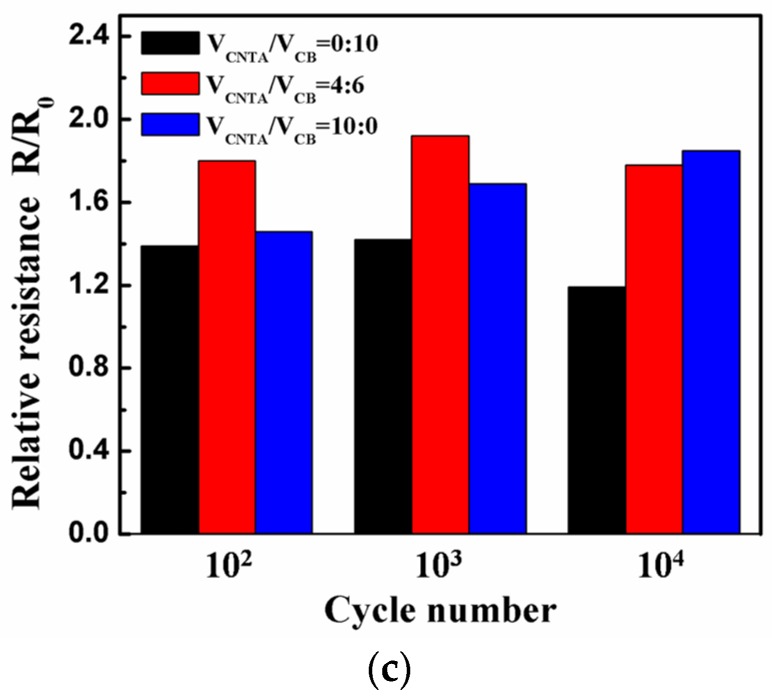
Fractional change of relative resistance *R*/*R*_0_ as a function of the stretch-recovery cycle number for composites at different volume fractions of fillers: (**a**) 1.5 vol %, (**b**) 3 vol % and (**c**) 6 vol %.

The effect of tensile strain history on the resistance is examined by fatigue tests. The specimens are stretched to a fixed tensile strain of 30% for 10^2^, 10^3^, and 10^4^ cycles. Figure 5 shows the fractional change of relative resistance *R*/*R*_0_ as a function of the stretch-recovery cycle number. For the majority of the composites, we can see from the bar graphs that the *R*/*R*_0_ increases with the increase of stretch-recovery cycle. For the CB/PMVS and CNTA/CB/PMVS composites with 6 vol % of fillers, as the stretch-recovery cycle increases, the *R*/*R*_0_ of the composites increases and then decreases, but the value of the *R*/*R*_0_ is still larger than 1.0. The values ranges of the *R*/*R*_0_ of CB/PMVS, CNTA/CB/PMVS, and CNTA/PMVS composites with 1.5 vol % of fillers are 1.60 to 1.84, 1.68 to 2.56, and 2.31 to 3.69, respectively. The value ranges of the *R*/*R*_0_ of CB/PMVS, CNTA/CB/PMVS, and CNTA/PMVS composites with 3 vol % of fillers are 1.50 to 1.63, 1.62 to 1.82, and 1.74 to 2.02, respectively. The value ranges of the *R*/*R*_0_ of CB/PMVS, CNTA/CB/PMVS, and CNTA/PMVS composites with 6 vol % of fillers are 1.19 to 1.42, 1.78 to 1.92, and 1.46 to 1.85, respectively. Larger fill content is propitious to smaller resistance change, representing a more stable filler network. Furthermore, the electric property of the CNTA/PMVS composites is less stable with the same filler contents. The PMVS composites with 3 and 6 vol % of fillers exhibit better electrical stability under different strain histories.

## 4. Discussion

### Relation of the Conductive Filler Network and the Strain-Sensing Behavior and Electrical Stability

**Figure 6:**
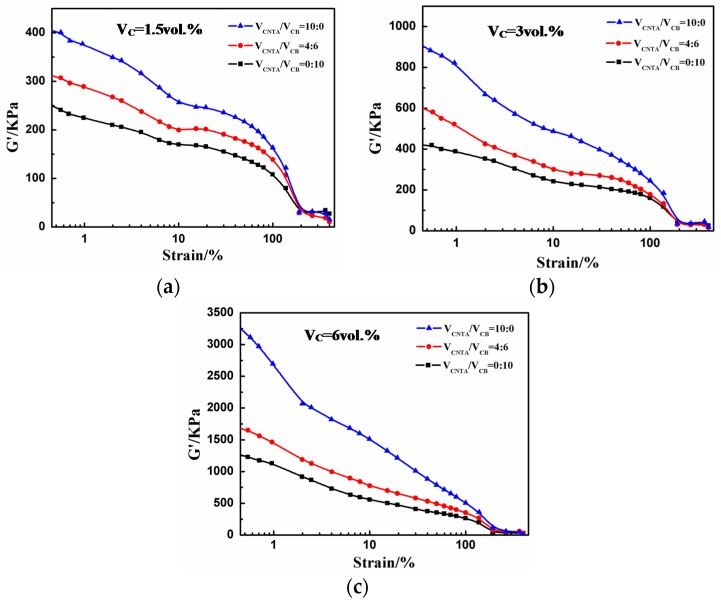
Storage modulus as a function of shear strain for composites at different volume fractions of fillers: (**a**) 1.5 vol %, (**b**) 3 vol % and (**c**) 6 vol %.

We used a RPA to study the conductive network of composites with different filler contents. Figure 6 shows that the storage modulus G’ decreases rapidly with the increase in shear strain for all the samples. This phenomenon is known as the Payne effect, which indicates the strength of the interaction among fillers in the matrix. The difference between the maximum and minimum of G’, named the modulus attenuation (ΔG’), is calculated according to the RPA curves to reflect the strength of the filler network in the rubber matrix more clearly, and a larger ΔG’ indicates a stronger conductive network. With the increase in filler content and the volume ratio of CNTA/CB, the Payne effect becomes stronger and it increasingly reflects fillers that come into contact each other, and ΔG’ increases from 235 to 3481 KPa. As a result, the conductive network becomes stronger.

**Figure 7:**
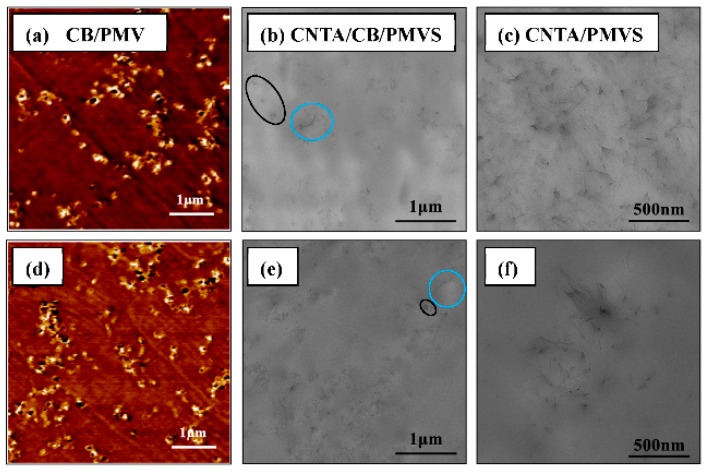
(**a**), (**d**) AFM adhesion micrographs of CB/PMVS composites with 1.5 vol % of CB; (**b**), (**e**) TEM micrographs of the composites with 1.5 vol % of CNTA and CB; (**c**), (**f**) TEM micrographs of the composites with 1.5 vol % of CNTA; images (**d**–**f**) refer to the corresponding composites after extension. The CNTAs and CB are shown in the blue and black oval area, respectively, in Figure 7b,e.

**Figure 8:**
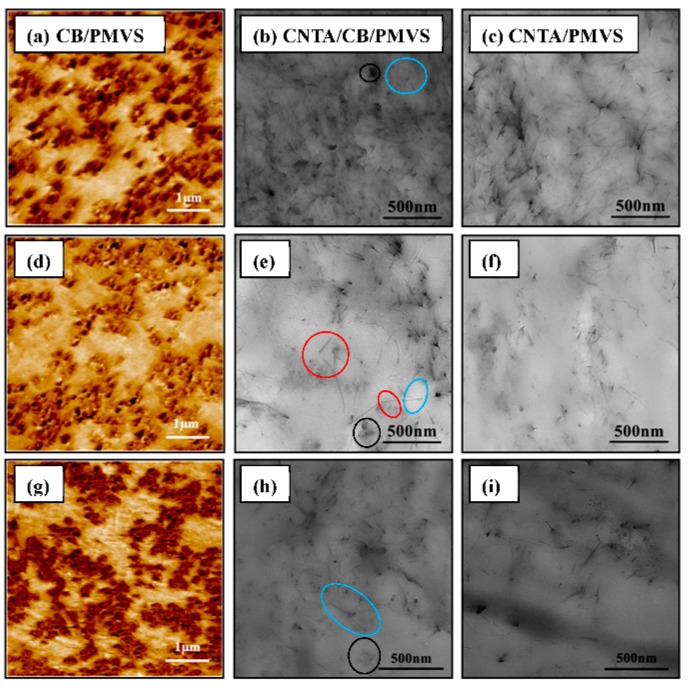
(**a**), (**d**), (**g**) AFM adhesion micrographs of CB/PMVS composites with 3 vol % of CB; (**b**), (**e**), (**h**)TEM micrographs of the composites with 3 vol % of CNTA and CB; (**c**), (**f**), (**i**) TEM micrographs of the composites with 3 vol % of CNTA; images (**d**–**f**) refer to the corresponding composites after extension; images (**g**–**i**) refer to the corresponding composites after 10^4^ repeated stretch-recovery cycles under fixed tensile strain 30%. The CNTAs and CB are shown in the blue and black oval area, respectively, in Figure 8b,e,h. The overlap between CNTs and CB is shown in the red oval area in Figure 8e.

**Figure 9:**
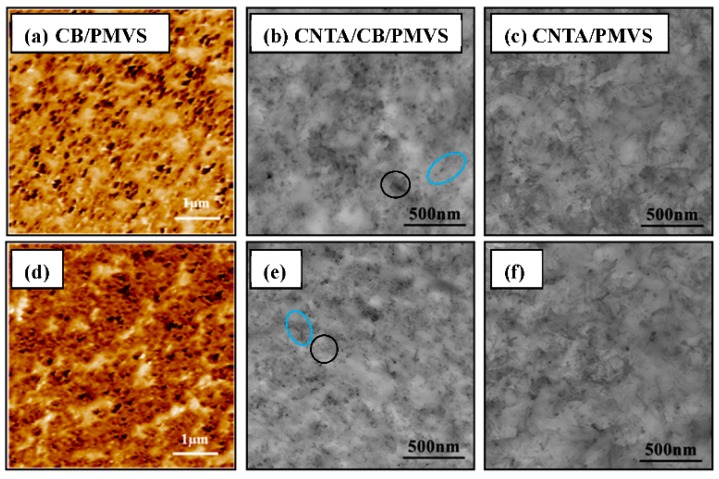
(**a**), (**d**) AFM adhesion micrographs of CB/PMVS composites with 6 vol % of CB; (**b**), (**e**) TEM micrographs of the composites with 6 vol % of CNTA and CB; (**c**), (**f**) TEM micrographs of the composites with 6 vol % of CNTA; images (**d**–**f**) refer to the corresponding composites after extension. The CNTAs and CB are shown in the blue and black oval area, respectively, in Figure 9b,e.

The morphology of filler dispersion in PMVS was observed with AFM and TEM micrographs. Figure 7, Figure 8, and Figure 9a–c represents the formation process of the conductive network. As shown in these micrographs, the fillers are mostly isolated at the filler content of 1.5 vol %, while, in composites of 3 vol % fillers, conductive fillers become partially aggregated and contact each other. When the filler volume fraction comes to 6 vol %, fillers begin to gather, and most fillers contact each other to form conductive network. This result is consistent with the Payne effect. As the filler content increases, a more complete filler network is formed. The formation of the network increases the conductivity of the composites. The length to diameter ratio of the CNTs is larger than that of CB, so the conductive network is stronger, and the conductivity is higher for the composites with higher volume ratio of CNTA/CB with the same filler content. As shown in Figure 7, Figure 8 and Figure 9b, in the CNTA/CB/PMVS composites, CNTA or CB fillers could contact each other to form conductive paths; in addition, CNTAs and CB also overlap each other to form the conductive network.

**Figure 10:**
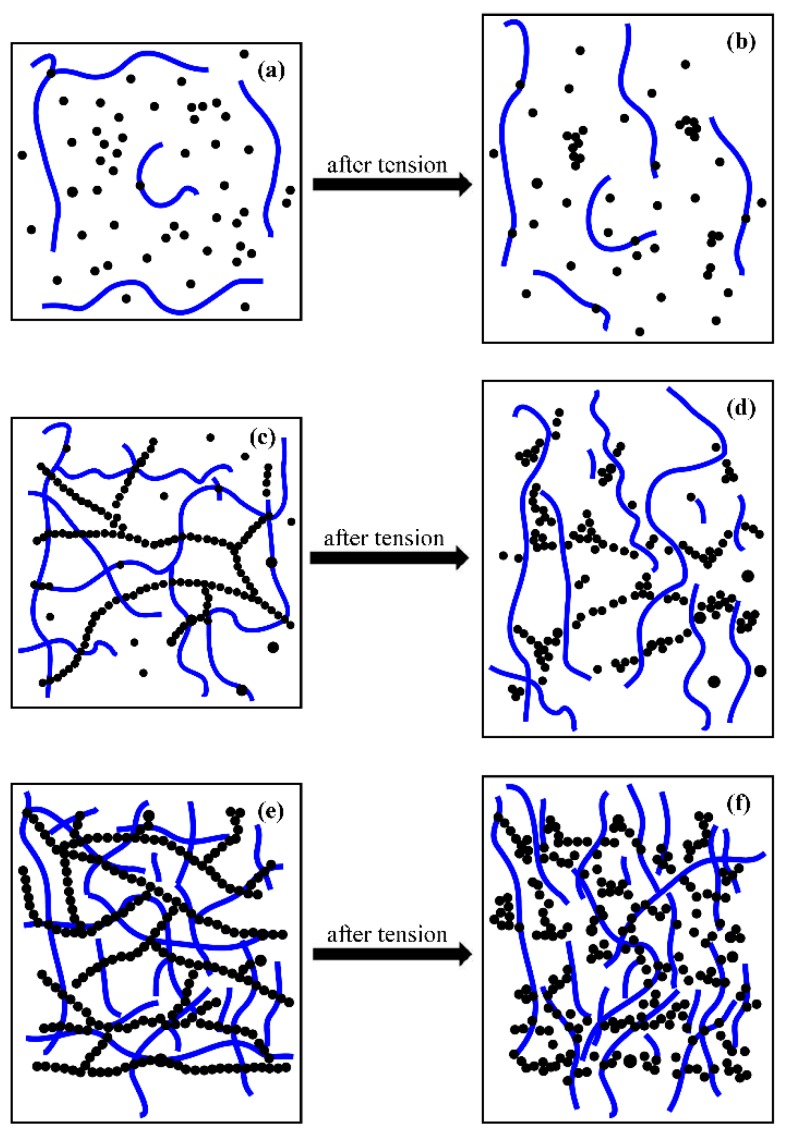
Schematics of filler networks: (**a**,**b**) 1.5 vol %; (**c**,**d**) 3 vol %; (**e**,**f**) 6 vol %; (**a**,**c**,**e**) initial condition; (**b**,**d**,**f**) after extension.

Figure 7, Figure 8 and Figure 9d–f represent micrographs of composites after the tensile fracture. Compared with the micrographs of unstrained samples, the dispersion of conductive fillers in the PMVS matrix is more nonuniform after the sample is subjected to tensile strain. An increase in the tunneling distance and loss in local contact between networks causes a decrease of conductive paths and an increase in resistance of composites. For CPCs during elongation, it is known that two phenomena occur simultaneously in the system: the breakdown of existing conductive paths and the formation of new conductive paths. These two phenomena compete with each other during the whole process. When the filler volume fraction is 1.5 and 3 vol %, the breakdown of conductive paths is more predominant than the formation of conductive paths in tension, the R/R_0_ increases exponentially with the strain, and the composites show high sensitivity, as depicted in Figure 2a,b. For the composites with 6 vol % of fillers, the number of the formation and breakdown of conductive paths are nearly the same, leading to the very slight increase of *R*/*R*_0_, and the composites show low sensitivity, as shown in Figure 2c. For the CB/PMVS composites, under the action of tensile stress, with increasing the strain, the distances between many CB particles grow, thus dominating the resistance of CB/PMVS composites. For the CNTA/PMVS composites, the large aspect ratio of CNTs is beneficial for the formation of the conductive network with conductive contacts and a more stable network structure. The difference in the microstructure of conductive network leads to higher sensitivity of composites with a lower volume ratio of CNTA/CB with the same filler content. The schematics of filler networks are shown in Figure 10. For all the composites, the dispersion of fillers becomes poor, and fillers are partially aggregated after tension. However, for the CNTA/CB/PMVS composite, due to the overlap between different fillers, the conductive network is still capable of being formed.

The repeatability of dynamic strain-sensing behavior is shown in Figure 4. When the filler volume fraction is 1.5 vol %, a fluctuation in *R*/*R*_0_ peak is observed, as the conductive network is fragile and unstable and could be damaged more easily under strain. When the filler volume fraction comes to 6 vol %, as a stronger and more stable conductive network is formed in the composite and the change of it is smaller under strain, the *R*/*R*_0_ becomes recoverable and the strain-sensing behavior remains repeatable. For the CB/PMVS and CNTA/PMVS composites with 3 vol % of fillers, a fluctuation in *R*/*R*_0_ peak is observed first, which is thought to be caused by the formation of additional conductive paths and the breakdown of original paths. However, the dynamic strain-sensing behavior of CNTA/CB/PMVS with 3 vol % of fillers is repeatable, which may be due to the synergistic effects of CNTAs and CB. The volume fractions of CB and CNTAs have just exceeded the percolation threshold, so CB and CNTs form a conductive network, respectively, contributing to a dual conductive network. Even after tension, the conductive network was still capable of being formed; thus, the composite shows high repeatability.

Figure 8g,h,i show micrographs of composites with 3 vol % of fillers after 10^4^ repeated stretch-recovery cycles under a fixed tensile strain of 30%. The redistribution of conductive fillers in the matrix after being applied to tensile strain causes an increase in resistance of the composites. As the adhesion between CNTs and matrix is relatively poor, CNTs are easier to aggregate, and the filler network of CNTs changes to be more nonuniform than that of CB after the fatigue tests, the electric property of the CNTA/PMVS composites is less stable than that of the CB/PMVS composites with the same filler contents.

## 5. Conclusions

To fabricate CPC strain sensors with tunable sensitivity, we used hybrid fillers of CNTAs and CB to control the conductive network structure in the PMVS matrix. The strain-sensing behavior and electrical stability are closely related to the conductive filler network, which strongly depends on the filler contents and the volume ratio of CNTA/CB. With the increase of filler contents and the volume ratio of CNTA/CB, the conductive filler network enhanced, and the sensitivity of composites decreased, while the repeatability and electrical stability under fatigue cycles of composites improved with the increase of filler contents.

For composites either filled with granular or fibrous fillers, the strain sensitivity is highest near the percolation threshold, but repeatability and electrical stability under fatigue cycles are the worst; on the other hand, at the region that far exceeds the percolation threshold, it is just the opposite. The percolation threshold of the CNTA composites is lower than that of the CB composites for a higher aspect ratio of CNTs. However, electrical stability under fatigue cycles of the CNTA composites is the worst, as the adhesion between the CNT and the matrix is relatively poor, and CNTs are easier to aggregate after fatigue tests. Therefore, it is difficult to achieve high sensitivity and repeatability and electrical stability simultaneously relying on a single CNTA or CB conductive network. The CNTA/CB/PMVS composite with 3 vol % content of fillers show high sensitivity (GF is 10 at 60% strain), high repeatability (the RSD of the max *R*/*R*_0_ value is 3.58%), and electrical stability under fatigue cycles (value range of *R*/*R*_0_ is 1.62 to 1.82) at the same time due to the synergistic effects of the CNTs and CB dual conductive network. For the volume fractions of CB and CNTAs that just exceeded the percolation threshold, both CB and CNTs formed a conductive network, respectively, to get a dual conductive network. It could still form a conductive network in the composite despite the destruction, but regeneration of the dual conductive network occurred under strain; therefore, the composite showed high repeatability and electrical stability. This study may provide guidance for the preparation of high performance CPCs for applications in strain sensors.

## Figures and Tables

**Table 1:** Values for measured electrical and mechanical properties of different composites. *V*c represents the total volume fraction of fillers in the composite, and *V*_CNTA_ and *V*_CB_ refer to the volume fraction of CNTAs and CB in the composite, respectively.

	Test items	Tensile strength (MPa)	Strain to failure (%)	Volume resistivity (Ω·cm)
Filler volume fraction and ratio				
*V*_C_ = 1.5 vol %	*V*_CNTA_/*V*_CB_ = 0:10	0.4	213	1.2 × 10^11^
	*V*_CNTA_/*V*_CB_ = 4:6	0.8	163	2.8 × 10^8^
	*V*_CNTA_/*V*_CB_ = 10:0	1.3	133	8.3 × 10^6^
*V*_C_ = 3 vol %	*V*_CNTA_/*V*_CB_ = 0:10	1.2	250	3,112.1
	*V*_CNTA_/*V*_CB_ = 4:6	1.8	180	854.0
	*V*_CNTA_/*V*_CB_ = 10:0	3.2	120	207.7
*V*_C_ = 6 vol %	*V*_CNTA_/*V*_CB_ = 0:10	2.9	259	12.2
	*V*_CNTA_/*V*_CB_ = 4:6	3.2	128	4.9
	*V*_CNTA_/*V*_CB_ = 10:0	5.4	78	3.3
